# Translation, Trans-Cultural Adaptation to Arabic, and Psychometric Testing of a Questionnaire Measuring Colorectal Cancer Knowledge, Perceptions, and Screening Practices among Average-Risk Population 

**DOI:** 10.31557/APJCP.2021.22.5.1537

**Published:** 2021-05

**Authors:** Fuad Hamdi Abuadas, Mohammad Hamdi Abuadas, Abdalkarem F. Alsharari, Zainab M Albikawi

**Affiliations:** 1 *Department of Nursing, College of Applied medical Sciences, Jouf University, Sakaka, Saudi Arabia. *; 2 *Faculty of Nursing, King Khalid University, Abha, Saudi Arabia. *

**Keywords:** Colorectal cancer, health belief model, tool validation, trans, cultural adaptation, Arabic version tool

## Abstract

**Purpose:**

Modifying, translating to Arabic, trans-culturally adapting, and testing the psychometric properties of colorectal cancer knowledge perception screening survey (CRCKPSS) to fit with Arabic culture to measure Jordanian average risk population’s health beliefs about colorectal cancer (CRC).

**Methods:**

A methodological cross-sectional design was employed to recruit a convenience sample of 460 average-risk Jordanian adults aged 50–75 years from the outpatient departments (OPDs) of two governmental hospitals in Jordan. The study was conducted in three phases: (a) Minimal modification of the CRCKPSS was undertaken. (b) Translation and transcultural adaptation of the modified version from English to Arabic were undertaken. (c) Validation of the trans-culturally modified Arabic version was performed.

**Results:**

Construct validity of the final trans-culturally modified Arabic version was evaluated by exploratory and confirmatory factor analysis, which yielded five factors. The total variance explained by all extracted factors was 83.4%. Cronbach’s alpha was applied separately for all five subscales and ranged between 0.94 and 0.98, indicating that the adapted version items have distinguishing consistency.

**Conclusions:**

Examining communities’ health beliefs regarding CRC is an important issue and requires a culturally valid and reliable scale. The modified Arabic version exhibited acceptable content, construct, convergent, and discriminant validity when used with the Jordanian average-risk population. Nurses and other health professionals can use it to assess beliefs about CRC and screening practices accurately. Moreover, the scale may be beneficial to other Arab countries, considering the diverse dialects within the Arab world.

## Introduction

Cancer prevalence and mortality rates reflect a major worldwide health problem (American Cancer Society, 2018). According to a report on global health statistics published by the World Health Organization, the annual number of deaths from cancer is expected to increase from 7.6 million in 2008 to approximately 13 million in 2030 (WorldHealthOrganization, 2012; AmericanCancerSociety, 2018). Globally, colorectal cancer (CRC) is ranked as the second most common cancer in females and the third most common cancer in males, with more than 500,000 deaths annually (Ferlay et al., 2014; Bray et al., 2018). Generally, the incidence rates for CRC are higher in developed, relative to developing countries. However, death due to CRC is more common in developing countries (52% of the total) relative to developed countries, reflecting a lower survival rate (Ferlay et al., 2014; Bray et al., 2018).

The precancerous polyps expand into invasive cancer over a long period (approximately ten years) (Siegel et al., 2020). This long period provides an opportunity to early detect and remove precancerous polyps, improving survival rates for CRC patients (Siegel et al., 2020). Screening is essential in the prevention process, as it could reduce the incidence of CRC and increase the likelihood of survival (AmericanCancerSociety, 2018). Despite the effectiveness of CRC screening and the availability of CRC screening tests, overall screening rates are low, and the pace of screening progression is slow (Bidouei et al., 2014; Ferlay et al., 2014; Reyes and Miranda, 2015; Bray et al., 2018; Abuadas and Abuadas, 2019). Several studies were conducted to improve understanding of the barriers that maintain low colorectal cancer screening rates (Bidouei et al., 2014; Almadi et al., 2015; Reyes and Miranda, 2015; Abuadas et al., 2018; Abuadas and Abuadas, 2019; Al-Hajeili et al., 2019). The perception of CRC risk in the broad community is highly important in ensuring a strong tendency toward screening and prevention. In several studies, most participants perceived themselves as good physically and believed that CRC screening was unnecessary (Bidouei et al., 2014; Almadi et al., 2015; Reyes and Miranda, 2015; Abuadas et al., 2018; Abuadas and Abuadas, 2019; Al-Hajeili et al., 2019).

The Health Believe Model (HBM) is considered one of the most famous theoretical models that improve understanding of health behavior by identifying participants’ attitudes as essential predictors of screening behavior (Champion and Skinner, 2008). HBM consists of several constructs. The first part is called perceived susceptibility, which represents the individual’s belief regarding the possibility of being at risk of developing a disease. The second part is called perceived severity, which represents an individual’s belief in a condition or disease’s seriousness. The third part is called perceived benefit, which represents the individual’s belief that the recommended action will decrease the risk of illness or reduce a condition’s seriousness. The fourth part is called perceived barriers, representing the individual’s beliefs regarding the potential cost and negative consequences of performing the recommended action (Champion and Skinner, 2008).

HBM has been used to investigate individuals’ health perceptions and practices related to diverse health situations. For example, the HBM primary constructs used by Victoria Champion to develop a specialized mammography screening scale to understand the motives underlying women’s self-examination of the breasts. Further, Champion stated that the scale could test other behaviors via substitution of words or phrases (Champion, 1999). The HBM has frequently been used to describe and predict cancer screening behavior, but it has rarely been used in CRC screening studies, particularly with Jordanian participants. The HBM has identified barriers and predictors and explained the relationships between them for many health behaviors in several studies involving various ethnic groups (Rimer, 2008). Green and Kelly adopted the Champion Revised Health Believe Model Scale (CRHBMS) (Champion, 1999) in 2004 to construct a reliable and valid instrument to determine factors related to CRC. Green and Kelly modified the CRHBMS to explore African-American participants’ knowledge, health beliefs, and screening practices regarding CRC (Green and Kelly, 2004). An accurate instrument’s cultural adaptation could empower healthcare professionals, particularly nurses, to assess CRC beliefs and screening practices accurately. Therefore, The HBM in this study will be used as a framework to translate, tans-culturally adapts, and validate The Colorectal cancer knowledge, perceptions, and screening survey (CRCKPSS).

*Study Purpose*

The primary study purposes were to (a) modify and translate the CRCKPSS to the Arabic language; (b) trans-culturally adapt the CRCKPSS to fit with Arabic culture, and (c) validate the culturally adapted Arabic version of CRCKPSS (psychometric properties).

## Materials and Methods

*Study design*

This cross-sectional methodological study was conducted in three phases. In phase 1, minimal modification of the CRCKPSS was undertaken. In phase 2, translation and transcultural adaptation of the modified CRCKPSS from English to Arabic were undertaken. In phase 3, the validation of the modified Arabic version of CRCKPSS (Psychometric proprieties) was performed. According to Polit and Beck (2017), cross-sectional methodological research is used to develop the reliability and validity of instruments, enabling researchers to collect a large quantity of data about the problem (Woo, 2017).

*Sample Size*

The researchers used a non-probability convenience sampling method to recruit 460 participants from the outpatient departments (OPDs) of two governmental hospitals in Amman-Jordan. The exclusion criteria were: (a) adults who had a previous diagnosis of CRC, (b) adults who had a family history of CRC, and (c) adults who were not able to talk or communicate with the researchers. Standards for the Selection of Health Measurement Instruments were used to calculate the sample size (Terwee et al., 2007). These consensus-based standards recommend a participant-to-variable ratio of at least 5 to 10 subjects per instrument item. The minimum number of estimated participants ranged from 210-420 based on a 42 item scale.

In the current study, the sample size was determined based on Westland (2010) statistical algorithm calculator website assuming a significance level (α) of 0.05, a medium effect size (f^2^ = 0.3), a power of 0.80, 5 latent variables, 42 observed variables would require a minimum sample size of 150 participants to detect the effect. 

*Data collection*

After obtaining permission to modify, use, and translate the CRCKPSS from the developers, the researchers received ethical approval from the nursing faculty ethics committee at the University of Jordan. Permissions from the Institutional Review Boards (IRB) at the two governmental Hospitals were also obtained before the beginning of this study. 

At the OPDs of hospitals, the researchers screened the potential participants for eligibility. After explaining the primary purposes, possible risks, and benefits of the study, verbal and written consent were obtained from all participants who agreed to participate. Participants were free to withdraw at any time, and they were informed that their withdrawal or refusal to participate would not affect the health services they received. Completing the survey by the participants took about 20-30 minutes. The researchers were available in a nearby place to answer any questions or concerns of any participant.

*Measurement scales*

*CRCKPSS*

The scale comprises three major sections: the first section, entitled “modifying factors,” comprises ten demographic items, two psychosocial items, and one structural item. The second section is composed of four subscales measured on a Likert scale (5-point). The subscales represent CRC susceptibility perception, CRC severity perception, Screening benefits perception, and screening barriers perception. In the second part, Cronbach’s α was 0.85 (Green and Kelly, 2004). The third part consists of 9 questions about CRC screening behaviors (Yes or No responses).

*CRHBMS*

Champion developed the CRHBMS based on the main concepts of the HBM. CRHBMS was revised, Champion assessed the psychometric properties, and it was used in a mammography screening study, “Revised Susceptibility, Benefits, and Barriers Scale for Mammography Screening” (Champion, 1999; Champion and Skinner, 2008). Champion proposed that the scales based on the HBM could be used with other behaviors, such as CRC, with substitution of words and phrases. Therefore, Champion’s scale was deemed appropriate for the measurement of health perceptions regarding CRC. The scale consists of five subscales and 28 items, measured using a 5-point Likert scale ranging from strongly agree to disagree strongly. The researchers used Only one subscale of the CRHBMS: the general health motivation subscale (7 items). Permission to use and modify the translated Arabic form of the revised Champion’s health belief model scale was obtained from the developers. The reliability coefficient alpha for the health motivation scale was .72 (Omran and Ismail, 2010).

*The procedure of modification, translation, and trans-cultural adaptation*

*Phase 1: Modification of the CRCKPSS*

Minimal modifications had been made for the first part of the CRCKPSS. The first part of the demographics section, the ethnicity item, was omitted due to the Jordanian study population’s homogeneity. Items concerning the history of CRC were also omitted, as this is one of the exclusion criteria. Whether participants have insurance coverage, measured on a “yes” or “no” scale, one demographic item was added. Besides, the participant was asked about the type of insurance they have. In the second part, the CRCKPSS does not include items that measure the health motivation construct of the HBM. Therefore, the health motivation subscale of the CRHBMS was added for the existing four subscales. All of the original subscales in the original version of CRCKPSS were included in the modified version. In brief, the second part of the modified version of CRCKPSS is composed of 5 subscales and 42 items measured on a 5-point Likert scale.

*Phase 2: (a) Translation of the modified version*

A panel consisting of two doctoral degree nurses specializing in community health nursing and two bilingual experts competent in English and Arabic translated the questionnaire. One of these individuals and one doctoral nurse translated the questionnaire from English to Arabic, and the others performed back translation. According to Waltz, Strickland, and Lenz [2010], back or double translation should involve two independent translators: one translates the items of the scale from the source English language to the target Arabic language, and the other translates the items from the target language to the source language (Waltz et al., 2010). No significant discrepancies were detected during the translation process.

*Phase 2: (b) Trans-cultural adaptation of the translated modified version*

The trans-cultural adaptation was performed by implementing two-round review processes. A panel of eight experts (two full professors, two associate professors in community health nursing, three assistant professors in oncology nursing, and one consultant oncologist) was contacted to evaluate the conceptual equivalence of the final modified Arabic version of CRCKPSS and suggest possible improvement in phrasing the translated items. In the first review round, a list of all item statements of the Modified Arabic version of CRCKPSS and information about the main HBM constructs’ theoretical and operational definitions were given to the experts. Based on the comments and suggestions, the Arabic version of the survey was modified. In the second review round, the same experts were given a 4- points’ ordinal scale to assess the items’ relevance to the construct being measured in the modified Arabic version. The 4- points’ ordinal scale rated as 4= extremely relevant, 3= fairly relevant, 2= slightly relevant, 1= non relevant. The expert rating agreement was then calculated using the Scale content validity index (S-CVI) and Item content validity index (I-CVI) (Polit and Beck, 2006).

*Phase 3: Validation of the modified trans-culturally adapted Arabic version of CRCKPSS*

The content validity of the modified trans-culturally adapted Arabic version of CRCKPSS was evaluated after the second review round by calculating I-CVI and S-CVI (See the results part). The construct validity of the five subscales measuring the HBM dimensions of the Modified trans-culturally adapted Arabic version of CRCKPSS was recognized using confirmatory factor analysis (CFA) and exploratory factor analysis (EFA). After completing CFA, discriminant and convergent validity were evaluated and illustrated in the result part. The researchers used the value of Cronbach’s alpha to evaluate the reliability coefficient of scale. 

*Data Analysis*

IBM Statistical Package for Social Sciences (SPSS) software (Version 21.0) and Analysis of Moment Structure (AMOS) software (Version 21.0) were used for all analyses. The data file was split randomly into two equal datasets (230 cases in each set); the first dataset was used for EFA (n=230), and the second dataset was used for CFA (n=230). Descriptive statistics were used to summarize the collected data and analyze the items in terms of average, standard deviation, and corrected item-total correlation. The reliability of the CRCKPSS questionnaire was assessed using internal consistency methods. While construct, convergent, and discriminant validity were assessed by EFA & CFA. Firstly, to understand the trans-culturally adapted modified Arabic version’s factor structure, 42 items were pooled and subjected to EFA. Factors were extracted using a principal axis factoring (PAF) analysis (Fabrigar et al., 1999). Promax rotation method was used to explore potential factor structures; the oblique rotation method will typically produce a greater level of simple structure since the current study factors are truly correlated (Warner, 2012). The statistical assumptions were assessed before performing data analysis. The researchers used the following criteria to establish the factor structure and retain items: (a) Kaiser-Mayer-Olkin (KMO) > .6; Kaiser–Meyer–Oklin (KMO) provides a method to compare the partial correlations to the zero-order correlations among pairs of variables (Kellar and Kelvin, 2013), (b) Significant value of Bartlett’s test of sphericity (p < 0.05); (Kellar and Kelvin, 2013)(c) eigenvalues ≥ 1, (d) clean factor loadings ≥ 0.40; factor loadings are considered clean if the absolute variation between loadings across different factors is greater than 0.20 (Nunnally, 1994). Secondly, CFA was subsequently applied to the data to examine the five-factor model’s construct validity; CFA was performed to determine whether the actual data set fit the hypothesized statistical model (Byrne, 2010). The following criteria were used to assess the measurement model fit: (a) factor loadings with a critical ratio (CR) higher than 1.96, demonstrating statistical significance; (b) relative chi-square index (*χ*^2^/df) or minimum discrepancy divided by its degrees of freedom (CMIN/DF) must be less than 2; (c) comparative fit index (CFI) and Normed fit index (NFI) must be more than 0.90; (d) goodness-of-fit index (GFI) and adjusted version (AGFI) must be more than 0.90; (e) root mean square error of approximation (RMSEA) must be less than 0.5; and (f) most standardized residual covariances between items less than two in absolute value (Hair et al., 2010).

## Results

*Socio-demographic characteristics*

The general socio-demographic characteristics of the participants are given in Table1. The mean participant’s age was 58.9 years (SD, 7.3 years), ranging between 50 and 75. Most study participants were male (55.7%), married (85.3%), had health insurance (69.6%), and had secondary education (46.5%). The majority of participants (71.5%) were unemployed (unemployed, housewife, retired).

*Content validity*

I-CVI was computed by dividing the total number of professional experts giving a rating of either 4 or 3 for the item on the total number of professional experts, while S-CVI was computed by taking the average proportion of items rated as 4 or 3 across all experts. The acceptable I-CVI value is > 0.78 for each item, and a value > 0.8 is acceptable for S-CVI (Lynn, 1986; Heavey, 2018). The content validity for all of the modified trans-culturally adapted Arabic version items ranged from 0.75 to 0.88, which are > 0.75 criterion, and for overall scale was 0.82, which is > 0.80 criterion. These results indicate that the Modified trans-culturally adapted Arabic version items have unique relevance to the measured constructs (Table 2).

*Construct validity*

Construct validity refers to the extent to which relationships among items included in a scale are congruent with the theory and the operational definition of concepts (Polit and Beck, 2013). The construct validity of the trans-culturally adapted modified Arabic version of CRCKPSS was recognized and established using EFA and CFA.

*Exploratory factor analysis*

In this study, The KMO measurement of sampling adequacy was 0.89, and Barlett’s test results were strongly significant (*χ*^2^ = 13401.87, df = 861, p < 0.001). Table 3 illustrates the EFA results. The preliminary factor analysis extracted five main significant factors. All item statements on each extracted factor were from the same original construct. The total variance explained by all extracted factors was 78.4%. The first factor accounted for 34% of the variance and loaded the thirteen items of barriers. The second factor accounted for 25% of the variance and represented all twelve items of the severity subscale. The third factor accounted for 9.15% of the variance and represented all seven motivation subscale items. The fourth factor accounted for 5.41% of the variance and represented all five benefits subscale items. The fifth factor 5, accounted for 4.87% of the variance and represented all five susceptibility subscale items. Loadings of items ranged from 0.59 to 0.97 in the rotated matrix pattern. The EFA yielded five mutually exclusive subscales having the same structure as the originally established measure.

*Confirmatory factor analysis*

Before performing CFA, the statistical assumptions and normality of the data were assessed. In the current study, the data’s multivariate normality was checked using IBM SPSS AMOS 21.0.0 software. The normality assumption is usually rejected if the kurtosis ratio’s value is greater than ±2 and the skewness value is greater than ±1(Nunnally, 1994). Skewness for all 42 items was less than ±1, and kurtosis for all 42 items was less than ±2. (Hair et al., 2010).

Table 4 shows the fit indices of the CRC Perceptions measurement model (42-item). The fit indices of the model were *χ*^2^ = 1512, df = 807, p < 0.001, GFI = 0.85, CFI = 0.91, AGFI = 0.82, and RMSEA = 0.070. The majority of the statistics disclosed that the moderately model fits well, except for GFI and AGFI. The majority of residual values were less than 2, except for the following large residual values: between Item 7 and Item 34 (2.81), Item 10 and Item 32 (2.03), Item 10 and Item 29 (-2.75), Item 10, and Item 28 (-2.03), Item 10 and Item 27 (-2.65), Item 10 and Item 26 (-2.02), Item 10 and Item 24 (2.12), and between Item 10 and Item 19 (2.25). Also, the weight of regression’s modification indices was examined to identify the parameter measures indicative of misspecifications and cross-loadings. However, the modification was not performed since a significant improvement of fit indices was not detected. Thus, the measurement model was accepted in its current shape.

**Figure 1:**
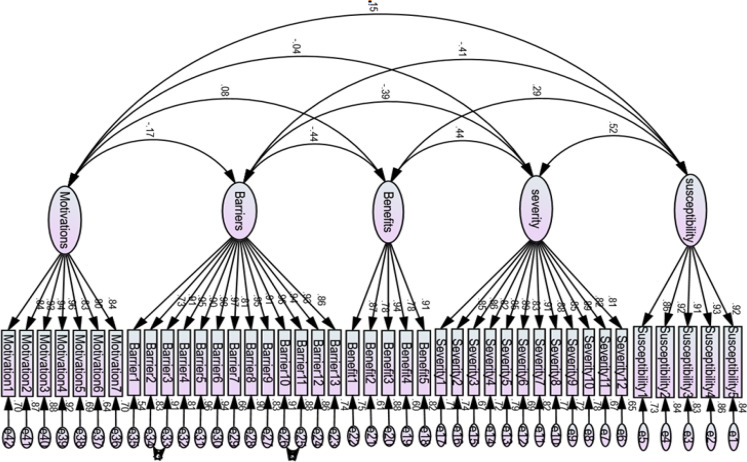
Acceptable and Significant Standardized Values (Factor Loadings) for All Items, Ranging from 0.73 to 0.96

The AMOS analysis structural model yielded five main constructs connected with double-headed arrows representing intercorrelations. Figure 1 shows acceptable and significant standardized values (factor loadings) for all items, ranging from 0.73 to 0.96; the critical ratios for all factor loadings were greater than 1.96, indicating statistical significance. The amount of variance (R^2^) attributable to each item ranged from 53% to 92%. The factor loadings for susceptibility items ranged from 0.86 to 0.93 with variance from 73% to 87%, severity items ranged from 0.81 to 0.91 with variance from 67% to 83%, benefits items ranged from 0.78 to 0.91 with variance from 61% to 83%, barriers items ranged from 0.73 to 0.93 with variance from 53% to 87%, and motivation items ranged from 0.80 to 0.96 with variance from 64% to 93%. The obtained results indicated that the original scale structure was supported for the 42-item. 

*Convergent and discriminant validity*

Hair et al., (2010), It is essential to evaluate and establish discriminant and convergent validity and construct reliability when completing a CFA. A few measures exist for establishing discriminant and convergent validity: average variance extracted (AVE), composite reliability (CR) (which represents the average percent of variation explained among the items in the same construct), maximum shared variance (MSV), and average shared variance (ASV). The AVE for each construct was used to evaluate convergent validity. The AVE was evaluated against its correlation with the other constructs; when AVE was above 0.50 and more extensive than the construct’s correlations with other constructs, then convergent validity was considered to be confirmed (Hair et al., 2010). To demonstrate discriminant validity, both MSV and ASV should be less than AVE for all constructs (Hair et al., 2010). Tables 5 show the measures used to establish convergent and discriminant validity. Convergent validity of the trans-culturally adapted modified Arabic version was established by assessing the AVE of all constructs; all AVE values were above 0.50 and more than the correlations with other primary constructs. Discriminant validity of trans-culturally adapted modified Arabic version was established by assessing MSV and ASV, both of which were found to be lower than the AVE for all scale constructs. 

*Reliability*

Internal consistency methods were utilized to assess the reliability of the scale. Cronbach’s alpha is considered the best technique used to estimate internal consistency (Polit and Beck, 2013). Cronbach’s alpha of more than 0.80 indicates high internal consistency, and about 0.7 is considered a sufficient value (Polit and Beck, 2013). The researchers used the following specific criteria to identify inappropriate and poorly functioning items : (a) if deleting the item results in an increase of more than 0.10 in the total reliability of the scale, or (b) a correlation of less than 0.30 between an item and the subscale score (Kellar and Kelvin, 2013). Cronbach’s alpha ranged between 0.92 and 0.97 for the extracted five subscales, indicating an acceptable value. The corrected item-total correlations were higher than 0.30 and ranged from 0.72 – 0.92. No item omission was made because the analysis of items showed that no item was predicted to augment the scale’s reliability if omitted significantly. The reliability coefficient of the total scale was 0.84. These results indicated that the trans-culturally adapted modified Arabic version items have unique consistency with one another. A summary of each subscale of Cronbach’s alpha coefficient values and other results were illustrated in Table 6. 

**Table 1:** Participants' Socio-Demographic Characteristics (N = 460)

Variables	Mean (SD)	Frequency	(%)
Age	58.9 (7.3)		
Gender			
Male		256	55.7
Female		204	44.3
Marital status			
Married		392	85.3
Widowed		43	9.3
Divorced/Single		25	5.4
Educational level			
Less than secondary		95	20.7
Secondary		214	46.5
Diploma		47	10.2
Bachelor's degree		89	19.3
Master's degree or higher		15	3.3
Currently work			
Yes		131	28.5
No		329	71.5
Income per month in JDS			
Less than 300		175	38.0
300–600		204	44.3
More than 600		81	17.6
Insurance			
Yes		320	69.6
No		140	30.4

JDS, Jordanian dinars; SD, standard deviation

**Table 2:** Experts Ratings of the Modified Trans-Culturally Adapted Arabic Version of CRCKPSS Items & Content Validity Indices

Item number	Number of Experts’ Non-Relevant Ratings (1 or 2)	Number of Experts’ Relevant Ratings ( 3 or 4)	I-CVI
Items (1, 2, 5, 11, 12, 13, 14, 18, 21, 22, 23, 25, 26, 29, 32, 34, 35, 38, 39, 40, 42)	1	7	0.88
Items (3, 4, 6, 7, 8, 9, 10, 15, 16, 17, 19, 20, 24, 27, 28, 30, 31, 33, 36, 37, 41)	2	6	0.75
S-CVI			0.82

**Table 3:** Exploratory Factor Analysis of the CRCKPSS (N = 230).

Factor	1		2	
Label	Barriers		Severity	
Item/	Item	Loading	Item	Loading
loading				
	11	0.96	5	0.97
	4	0.94	6	0.92
	6	0.93	3	0.91
	10	0.92	7	0.91
	3	0.91	9	0.9
	2	0.86	8	0.89
	5	0.84	4	0.89
	9	0.82	12	0.82
	12	0.82	10	0.82
	7	0.8	2	0.81
	8	0.76	11	0.81
	13	0.73	1	0.77
	1	0.59		
Eigen value	14.28		10.5	
Variance explained	34%		24.96%	

**Table 4:** Goodness-of-Fit Indices for Trans-Culturally Adapted Modified Arabic Version of CRCKPSS Model (n=230)

Fit Indices	Model Fit Criteria	Colorectal Cancer Perceptions Survey Results
CMIN/DF or (χ^2^/df)	CMIN/DF ≤ 2	( 1512.45/807) = 1.86
TLI	TLI > 0.90	0.88
RMSEA	RMSEA ≤ 0.06	0.07 (LO 90 = 0.067, HI = 0.073, PCLOSE 0.06)
GFI	GFI > 0.90	0.85
AGFI	AGFI > 0.90	0.82
CFI	CFI > 0.90	0.91
SRMR	SRMR < 0.08	0.08

χ^2^/df, relative chi-square; GFI, goodness of fit index; AGFI, adjusted GFI; CFI, comparative fit index; SRMR, standardized root mean square residual; TLI, Tucker Lewis index; RMSEA, root mean square error of approximation

**Table 5:** Inter-Correlations of Subscales, Convergent and Discriminant Validity Measures for Trans-Culturally Adapted Modified Arabic Version of CRCKPSS (N = 230)

	Subscales	CR	AVE	MSV	ASV	1	2	3	4	5	Convergent validity CR > AVE AVE > 0.50	Discriminant validity MSV < AVE ASV < MSV
1	severity	0.97	0.728	0.272	0.155	0.853					Yes	Yes
2	susceptibility	0.958	0.821	0.272	0.135	0.522**	0.906				Yes	Yes
3	Benefits	0.932	0.733	0.194	0.118	0.436**	0.285**	0.856			Yes	Yes
4	Barriers	0.984	0.828	0.194	0.136	-0.395**	-0.405**	-0.441**	0.91		Yes	Yes
5	Motivations	0.959	0.771	0.028	0.015	-0.037	0.149*	0.082	-0.168*	0.878	Yes	Yes

Square root of AVE in bold on diagonals; * p < .01; ** p < .001 (two-tailed)

**Table 6:** Reliability Coefficients of Trans-Culturally Adapted Modified Arabic Version of CRCKPSS Subscales (N = 230)

Subscale	Number of items	Cronbach's alpha	Range of corrected item-total correlations	Mean	SD
Susceptibility	5	0.92	0.76– 0.82	2.91	1.13
Severity	12	0.97	0.82- .090	3.20	1.28
Benefits	5	0.95	0.89- 0.93	3.49	1.38
Barriers	13	0.94	0.74- 0.91	3.34	1.26
Health motivation	7	0.96	0.83- 0.92	3.12	1.47
Overall Scale Cronbach's alpha (0.84)					

SD, standard deviation

## Discussion

Many research studies have been conducted to understand the individuals ‘CRC health beliefs and their screening practices in a different population (Omran and Ismail, 2010; Christou and Thompson, 2012; Ahmad, 2014; Ahmad et al., 2015; Almadi et al., 2015; Alsanea et al., 2015; Abuadas and Abuadas, 2019; Al-Hajeili et al., 2019). A reliable and valid instrument is necessary to recognize and understand such health beliefs. The current study is the first study that describes instrument modification, adaption, and validation processes for instruments measuring CRC health beliefs and screening practices in Arabic culture. The study results suggest that the adapted, modified Arabic version is a reliable and valid instrument to assess the CRC health perceptions and screening practices in Arabic culture and has an adequate factor structure based on HBM’s primary constructs. 

All of the trans-Culturally adapted modified Arabic version subscales were found to be mutually exclusive and internally consistent. Cronbach’s α for all subscales was excellent and ranged from 0.94 and 0.98. The Cronbach’s α obtained were higher than those of Green and Kelly (2004) and Omran and Ismail (2010). Furthermore, the obtained Cronbach’s α was roughly similar to previous studies conducted in Turkey (Ozsoy et al., 2007). The average item-total correlation and average interitem correlation were also high.

We found that the trans-Culturally adapted a modified Arabic version as content valid after the expert panel’s excellent agreement on the relevance of items. Content validity appeared sufficiently high. I-CVIs of All items were acceptable and ranged from 0.75 to 0.88, while the total-scale S-CVI value was (0.82). These results showed that the scale has unique relevance to the measured constructs. The indices of content validity obtained in the current study were roughly similar to previous studies conducted based on the HBM constructs (Green and Kelly, 2004; Omran and Ismail, 2010; Lee et al., 2017)

Two distinct conceptual processes (CFA and EFA) were utilized to test the trans-Culturally adapted modified Arabic version’s construct validity. EFA results identified five factors and all of the items in each subscale (CRC susceptibility, CRC seriousness, barriers of CRC screening, and CRC screening benefits) loaded on their respective subscales as in original CRCKPSS (Green and Kelly, 2004). Similarly, all of the health motivation subscale items are loaded on the same respective subscale of the original CRHBMS (Champion, 1999; Omran and Ismail, 2010). Furthermore, CFA results indicated that the 42-items of the trans-Culturally adapted modified Arabic version support the original scales structure and fit the data significantly. The results of EFA and CFA are confirming the construct validity of the trans-Culturally adapted modified Arabic version. Previous studies demonstrated similar results in that all selected subscale items loaded significantly on their respective original subscales in both conceptual factor analysis processes (Champion, 1999; Green and Kelly, 2004; Champion and Skinner, 2008; Lee et al., 2017). However, the health motivation and severity subscales items were loaded on two factors in other studies investigating the health belief model’s same constructs regarding breast cancer (Mikhail and Petro-Nustas, 2001; Parsa et al., 2008). Besides, the construct validity of the trans-Culturally adapted modified Arabic version was strongly supported through convergent and discriminant validity examinations. To date, the current study is unique in that it examined convergent and discriminant validity concerning CRC. The current study’s findings indicated that HBM constructs concerning CRC could be measured with a substantial amount of convergent and discriminant validity using 5-point Likert scale items. The culturally adapted modified Arabic version exhibited acceptable content, construct, convergent, and discriminant validity when used with the Jordanian average-risk population.

Healthcare professionals can use the culturally adapted, modified Arabic version, particularly nurses, to assess CRC beliefs and screening practices accurately. Such an evaluation is essential to identify the primary learning needs and formulate educational programs specifically tailored to target the main misunderstanding and faulty perceptions. Also, the scale could be used to test the efficiency of culturally sensitive intervention strategies. Moreover, the scale may be beneficial to other Arab countries, considering the diverse dialects within the Arab world.

The following limitations must be considered in the current study findings. The study recruited the sample from a limited number of clinics from only two governmental hospitals using a convenience sampling procedure. Therefore, the study sample may not be representative of all Jordanian average-risk populations. Besides, using a self-report scale could have introduced biases in the study, such as recall bias and social desirability biases. Despite the limitations, this study addressed critical issues concerning developing a culturally sensitive scale to measure health perceptions regarding CRC and screening practices. Continued work on testing and refining the culturally adapted modified Arabic version on varied population groups is necessary and recommended.

In conclusion, a reliable and valid instrument is essential in identifying and understanding the target group’s health perceptions regarding CRC and screening practices. The culturally adapted modified Arabic version of CRCKPSS is appropriate for assessing individuals’ health beliefs about CRC and their Arabic Jordanian culture screening practices.

## Author Contribution Statement

All authors contributed to the study conception, design, data collection, analysis, and interpretation of the results. 
